# Data processing pipeline for managing enteric methane emissions from sniffer: Application on dairy farming systems

**DOI:** 10.3168/jdsc.2026-1018

**Published:** 2026-05-20

**Authors:** Chiara Rossi, Coralia I.V. Manzanilla-Pech, Giampiero Grossi, Birgit Gredler-Grandl, Nicola Lacetera, Andrea Vitali

**Affiliations:** 1 Department of Agriculture and Forest Sciences, University of Tuscia, Viterbo, Italy, 01100; 2 Animal Breeding and Genomics Center, Wageningen Livestock Research, Wageningen University & Research, Wageningen, the Netherlands, 6708 PB

## Abstract

Accurate quantification of enteric methane (CH_4_) emissions from ruminants is essential to support global strategies aimed at mitigating GHG outputs from the livestock sector. Among the available in vivo techniques, the sniffer method offers a noninvasive and cost-effective solution for large-scale monitoring, but lacks standardized procedures for data processing and validation. This study presents a pipeline for processing sniffer-derived raw data to estimate individual CH_4_ and carbon dioxide (CO_2_) emissions from dairy animals. The procedure was tested on 3 commercial farms in central Italy, consisting of 141 monitored animals and 47,018 milkings, each rearing a single breed: Holstein-Friesian cows, Brown Swiss cows, or Mediterranean buffaloes. The pipeline integrates measurements from sniffers with automatic milking system data and includes procedures for time alignment, data cleaning, background gas correction, and CH_4_ peaks detection. Individual daily CH_4_ production was estimated considering the amount of CO_2_ predicted and the CH_4_:CO_2_ ratio method. Methane emissions averaged 416.7 ± 74.9 g/d for Holstein-Friesian and 502.1 ± 158.4 g/d for Brown Swiss cows, differing by approximately 10% from the Intergovernmental Panel on Climate Change (IPCC) Tier II model estimates. For buffaloes, average CH_4_ emissions were 279.0 ± 97.5 g/d, which were consistent with the emission factor estimated by Tier II of the IPCC. The proposed pipeline provides a harmonized and replicable framework for processing sniffer data, improving the reliability and comparability of enteric CH_4_ and CO_2_ emission measurements across ruminant species and farming systems.

## Highlights

• Sniffers effectively monitor enteric gas concentrations in dairy farming systems.
• Processing of sniffer data is needed to quantify individual enteric emissions.
• Harmonized procedures of sniffer data can improve repeatability among studies.

## Graphical Abstract

**Figure:**
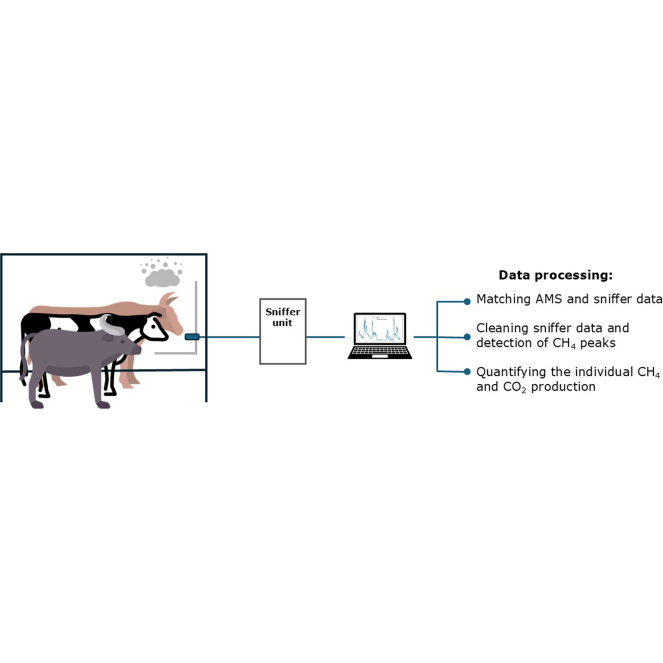
**Summary:**Monitoring ruminants' enteric emissions with sniffers is expanding worldwide to assess individual emissive levels and support CH4 reduction programs. However, it is necessary to provide accurate data-processing procedures for the quantification of enteric CH4 emissions from raw sniffer data. This study aimed to provide a guided and replicable pipeline to estimate individual enteric emissions with a step-by-step approach tested on 3 different dairy systems. AMS = automatic milking system. The graphical abstract was designed by the authors using Microsoft PowerPoint (Version 2604 Build 16.0.19929.20136) and resources from Flaticon.com. Icons made by Icongeek26, Yuluck, and Freepik from www.flaticon.com.

Sniffers are increasingly used worldwide as noninvasive and cost-effective systems for monitoring individual enteric emissions and supporting breeding programs. Sniffer systems continuously sample air through an inlet tube positioned in the feed trough of the automatic milking system (**AMS**) or automatic feeder and measure methane (CH_4_) and carbon dioxide (CO_2_) concentrations in the collected air (Lassen et al., 2012). Because the sniffer operates as a stand-alone system for data acquisition, procedures are required to align gas concentration data with animal visits and estimate individual emissions (Milkevych et al., 2022). A common concern among sniffer users is the reliability of recorded data, given the strong influence of barn CH_4_ and CO_2_ concentrations and the proximity of the animal's muzzle to the sampling point (Huhtanen et al., 2015). Different approaches have been proposed to improve consistency of sniffer data, including addressing clock-synchronization issues with AMS and detecting hardware faults (Milkevych and Villumsen, 2025; van Breukelen et al., 2025). To quantify animal emissions, the CH_4_:CO_2_ ratio method has been widely adopted. This method uses the ratio of gas concentrations detected by sniffer and estimates animal's individual CH_4_ emissions based on its predicted CO_2_ production (Madsen et al., 2010). This study proposes a guided pipeline that leads sniffer users through a step-by-step approach, from raw data processing to the quantification of individual CH_4_ and CO_2_ emissions. It includes criteria for combining sniffer data with data from the AMS and procedures to manage the sources of inaccuracy in sniffer recordings. To show the implementation of the pipeline and differences in emission patterns, this study presents case studies from 3 commercial dairy farms. Due to the lack of in vivo systems measuring emissions (g/d) from the same animals, individual-level sniffer emissions were compared with herd-level estimates derived from Intergovernmental Panel on Climate Change (IPCC, 2019) Tier II model. Sniffers (MooLogger, Tecnosens SpA, Italy) were installed on 3 commercial dairy farms located in central Italy. Two farms operated under intensive management system rearing Holstein-Friesian (**HF**) cows and Mediterranean buffaloes (**MB**), respectively. The third farm was organic and reared Brown Swiss (**BS**) cows. A total of 141 lactating animals were monitored twice during 2024, with each monitoring session lasting approximately 1 mo and occurring in different periods of the year. Animals on the 3 farms were fed a partial mixed ration (**PMR**; Table 1), with daily feed residues estimated at between 0% and 5%. Additionally, animals received an extra amount of concentrate through the AMS. These amounts varied according to production levels and averaged around 4 kg/d for HF cows, 1 kg/d for BS cows, and 2.5 kg/d for MB. Finally, BS cows had free access to alfalfa pasture for 5 h/d. Sniffers were installed within the AMS, which differed among farms. The HF farm was served by a Lely Astronaut A4, the BS farm used a Gea Dairy Robot R9500, and the MB farm was equipped with a DeLaval VMS V310. Differences among AMS were mainly related to feed bin design; however, sniffer installation was kept as consistent as possible across farms to ensure comparable conditions. The installation involved a 5 to 7 tube connecting the sensor box to the feed bin, depending on available space, and 2 inline filters to remove coarse and fine particles. The sampling point was positioned according to Garnsworthy et al. (2012). In the Lely and DeLaval AMS, the tube was placed adjacent to the concentrate delivery chute and hidden behind the front panel, with the inlet positioned vertically near the panel edge and the opening of the feed bin to capture exhaled breath. In the GEA AMS, the tube was hidden behind the rotating feed bin, with the sampling point near the unused liquid dispenser opening. Gas was sampled at an air flow rate of 1 L/min, and CH_4_ and CO_2_ concentrations were measured at 1-s intervals using nondispersive infrared sensors. Sniffer calibrations were performed to set zero and the span of the sensors. The zero-point was calibrated at the beginning of each monitoring period and every 2 wk by flushing compressed nitrogen (99.999%). The span calibration used to set the upper limit of the sensor scales was performed only at the beginning of the monitoring periods using a gas mixture containing 0.8% CH_4_ and 4% CO_2_, with nitrogen as balance gas (Calgaz Ltd., Staffordshire, UK). Sniffer and AMS data were processed to estimate individual emissions both in absolute terms (g/d) and intensity, defined as the amount of CH_4_ produced per unit of milk corrected for its energy content. The procedure was automated using Python v3.12 (https://www.python.org/), and the description of the sequential operations is provided in the following list.

1. **Matching AMS and sniffer data:**Sniffers lack animal identification systems and continue to log data even during AMS idle periods. To ensure that sniffer data corresponded to actual animal presence, AMS visit timestamps were aligned with CH_4_ and CO_2_ measurements (Difford et al., 2016; Milkevych et al., 2022). First, the AMS dataset was filtered by discarding animals with zero milk production or rejected because they did not spend sufficient time near the sniffer sampling tube. In all case studies, both sniffer and AMS were internet-connected, and their internal clocks were synchronized to a network time protocol server. Sniffer clocks were automatically set to GMT/UTC (UTC+00:00), whereas AMS recorded local time (CET/CEST). Thus, sniffer timestamps were adjusted for local time zone and daylight saving to align with AMS records. Once AMS and sniffer datasets were combined, the outcome presented CH_4_ and CO_2_ recordings only ascribable to milking sessions, including AMS information of individual-level data (e.g., productivity, DIM, number of lactation).
2. **Cleaning sniffer data and detection of CH_4_ peaks:** Sniffer measurements may be influenced by barn emissions and diurnal changes, as well as by sensor drift, instrument malfunctioning, and installation-related configuration (Hammond et al., 2016). Alongside calibration and maintenance procedures aimed at reducing sensor drift and equipment malfunctions, a set of criteria was defined to enhance the accuracy of sniffer measurements. First, the Fourier method was used to model diurnal fluctuations and adjust CH_4_ and CO_2_ data (L⊘vendahl et al., 2024). Then, outliers and negative values commonly resulting from sensor errors during high-frequency recording were identified and removed. Outliers were detected by using the third SD method (Martinez-Boggio et al., 2025) and filtered from milking recordings along with negative data. Enteric CH_4_ derives from eructation events, which appear on the sniffer trace as a rapid rise followed by an exponential decay (Wang et al., 2023). The CH_4_ irregular pattern enabled the background removal during milkings, as proposed by van Engelen et al. (2018). Background concentrations were estimated as the lowest 10% of measurements within each visit. This threshold was selected through a sensitivity analysis using the 1%, 5%, 10%, and 15% lowest values. For each threshold, the Pearson correlation coefficient was computed and squared to obtain the coefficient of determination (R^2^). The 10% threshold provided the highest average fit (mean R^2^ = 0.925). Each CH_4_ measurement was baseline-corrected by subtracting the estimated background. In contrast to CH_4_, emitted CO_2_ derives mostly from respiratory events and typically shows several stable upper periods associated with respiration events and a limited number of low values (McGinn et al., 2021). For this reason, lower thresholds were evaluated to define the lowest points within each visit. A sensitivity analysis was performed using the 0.5%, 1%, 1.5%, 2%, and 3% lowest values to define the CO_2_ background. For each threshold, the Pearson correlation coefficient was calculated and squared to obtain R^2^. The highest average correlation (mean R^2^ = 0.994) was obtained with the 1% threshold. Then, CO_2_ records were baseline-corrected accordingly. Sniffer recordings are subject to inlet-to-sensor transport lag due to the tubing length. As a result, lag time can cause carryover between sequential AMS visits. To address this issue, the delay was empirically measured via a step-input test (gas released in the feed bin) to quantify the sensor's response time. Several tests were performed at farms throughout the year to account for varying environmental conditions. The average lag time was used to define the threshold, resulting in 25 s, and the corresponding CH_4_ and CO_2_ measurements were removed from each milking. However, the pipeline can be customized by specifying the value obtained from the step-input test. The CH_4_ peaks observed during milkings reflect animal eructation events. The number of peaks per visit was used as an indicator of animal's proximity to the sampling inlet, ensuring reliable sniffer data (Hardan et al., 2022). The *find_peaks* function from *scipy.signal* package (SciPy, http://www.scipy.org) was used to detect CH_4_ peaks during milking. Briefly, the function identifies local maxima in a series and allows to filter using parameters such as minimum peak height and minimum distance between peaks. Based on Bell et al. (2020) and testing constraints to reduce the surrounding noise, criteria were defined to standardize the identification of eructated peaks in both cows and buffaloes.
  a. Peak height: Different thresholds were used for buffaloes and cows to identify eructation spikes due to differences in peak shape. For buffaloes, values greater than twice the mean of the CH_4_ series were considered, whereas for cows, values above the mean were sufficient. The CH_4_ signal in buffalo visits showed narrow, sharp peaks, whereas cow CH_4_ signals showed regular peaks with well-defined amplitudes (Figure 1). The need for different peak-height thresholds was supported by CH_4_ data distribution within visits. The average skewness and kurtosis of CH_4_ were 2.194 and 5.651 for buffaloes and 1.118 and 0.701 for cows. In both cases, most data were concentrated at low values, with the right tail corresponding to eructation events. However, the much higher skewness and kurtosis in buffaloes indicates a more asymmetric and sharp distribution, heavily influenced by low concentrations. Consequently, using the mean to define peaks in buffalo data would result in detecting noise together with true eructation events.

**Figure 1:**
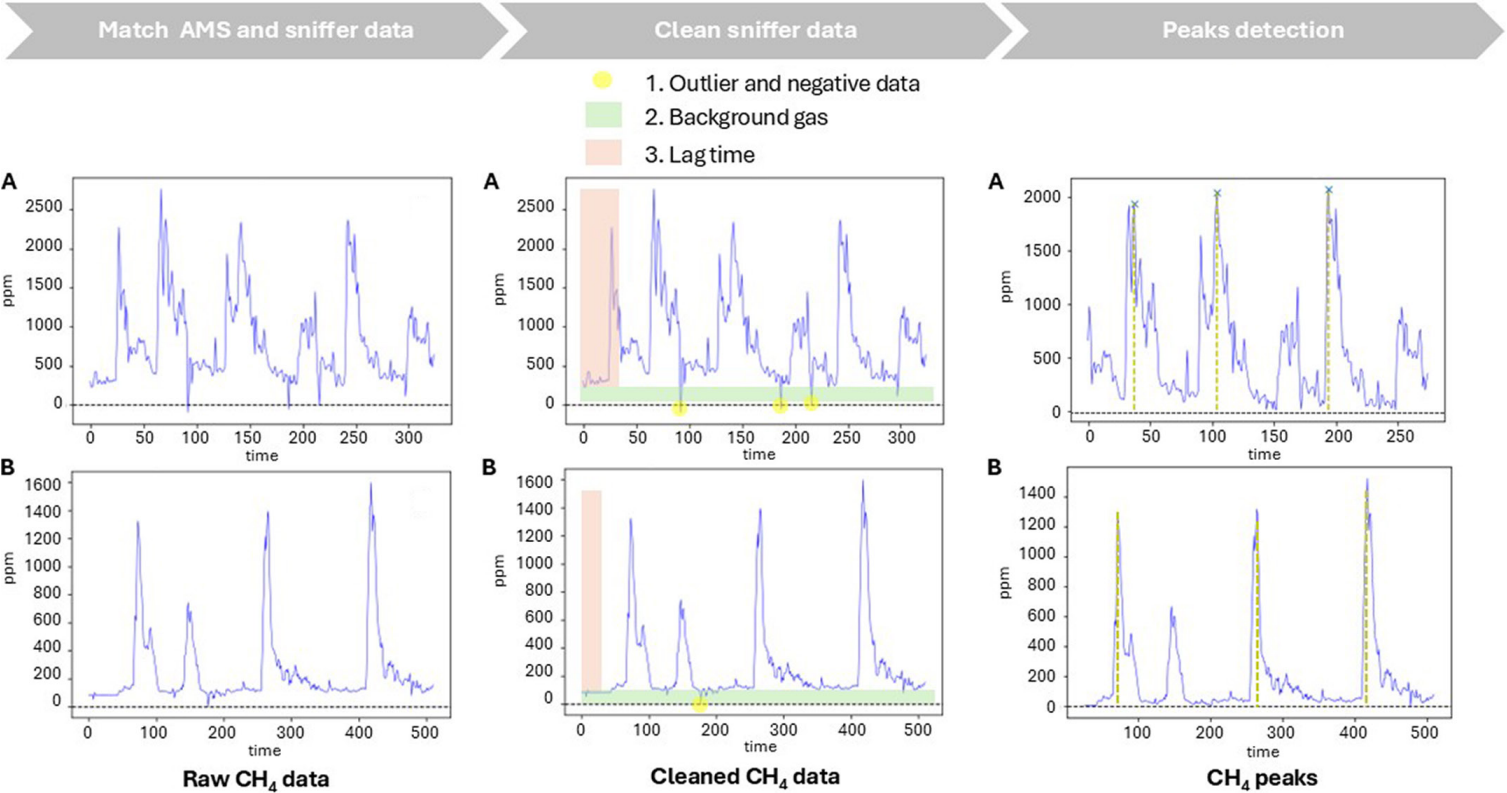
Workflow of sniffer data processing during cow (A) and buffalo (B) milking visit.
  b. Peak width: Spans covered at least for 3 s at half-height of the peak to exclude inaccurate peaks formed by isolated high data points.
  c. Distance between peaks: The minimum was 35 s. Given the distinctive pulse-release pattern of CH_4_ peaks, with eructation events occurring every 45 to 60 s (Garnsworthy et al., 2012; Huhtanen et al., 2015), the apex did not appear at the midpoint of the peak but rather at its onset. For this reason, the 35-s interval represented the tail portion of the peak before the start of the next one.
  Peak counts were recorded for each milking, and milkings with at least 3 peaks were retained for CH_4_ and CO_2_ quantification (Bell et al., 2020). The outcome of the cleaning procedure was a dataset of daily CH_4_ and CO_2_ concentrations per animal, calculated as the average of cleaned sniffer measurements per day. The cleaning procedure applied within a milking visit is illustrated in Figure 1.
  3. **Quantifying the individual CH_4_ and CO_2_ production:**Starting from the cleaned dataset, individual CH_4_ and CO_2_ production was quantified. Individual CO_2_ emission in grams per day was predicted using the “On-farm model” developed by Kjeldsen et al. (2024). For HF cows, breed-specific values were used, whereas in the absence of specific values for BS cows or buffaloes, the values for “Others/crossbreeds” were used. The “On-farm model” is based on animal information, which includes BW, milk yield, DIM, and parity. Table 1 reports input information employed in the model. Energy-corrected milk, which is also required in the “On-farm model,” was calculated using the equations developed by Campanile et al. (2003) for buffalo milk and by Sjaunja et al. (1990) for cow milk. Daily CH_4_ emissions were determined by applying the CH_4_:CO_2_ ratio to the calculated daily CO_2_ production. Because the CH_4_:CO_2_ ratio is defined on a volumetric basis (L/d), the calculated grams of CO_2_ were converted in liters by multiplying by CO_2_ as L/g at standard temperature and pressure (**STP**, 273.15 K and 101.325 kPa) as follows: CO_2_ (L/d) = CO_2_ (g/d) × 0.509 (L/g).

**Table 1:** The partial mixed ration (PMR) composition and input information employed in the “On-farm” model of the 3 case studies

Item	Variable	Farm		
		HF	BS	MB
Monitored animals, n		69	36	36
Animal characteristic	BW,^1^ kg	650	700	550
	Milk production, kg/d	35.0 ± 8.5^2^	19.7 ± 4.7	7.3 ± 3.4
	DIM, n	172 ± 124	309 ± 157	235 ± 107
	Parity, n	2.3 ± 1.3	2.8 ± 1.6	1.3 ± 0.6
Diet composition	PMR, kg DM/d	22.7	22.6	17.0
	Grass hay	4.8	6.8	3.5
	Alfalfa hay	4.0	4.4	1.3
	Grass silage	—3	3.2	2.9
	Sorghum silage	2.9	—	—
	Sorghum grass	—	—	0.4
	Byproduct feeds	1.2	4.9	2.7
	Corn flour	6.7	2.2	3.8
	Soybean flour	—	—	1.0
	Barley flour	—	1.1	—
	Supplements	3.2	—	1.4

1 Body weights were assumed based on reference values for HF (Spek et al., 2022) and BS (Frey et al., 2018) cows and MB (Sabia et al., 2014).

2 Reported variability (values after the ±) represent SD.

3 Ingredient not included in the diet.

After applying the CH_4_:CO_2_ ratio, the resulting CH_4_ in liters per day was reconverted into grams per day, considering the CH_4_ density at STP conditions (0.715 g/L). As recommended by Bhatta and Enishi (2007), STP conditions were chosen to convert daily gas production for comparison with gas measurements by other techniques. The STP correction was consistently applied throughout all data conversions. Case studies data reported in Table 1 were also employed to estimate enteric CH_4_ emissions at herd-level by applying the Tier II methodology defined by IPCC (Equation 10.21 in IPCC, 2019), which is based on daily gross energy intake and CH_4_ conversion factor (Ym). The Ym values were estimated using a linear equation developed by Rossi et al. (2023). Based on the forage content of the diet, Ym values were estimated at 6.02 for HF cows, 6.58 for BS cows, and 6.24 for MB. Emissions calculated using the IPCC (2019) methodology were compared with average individual-level CH_4_ emissions measured by sniffer. This comparison benchmarks animal emissions from the same farms using 2 different approaches; therefore, it is not intended for pipeline validation purposes. Pipeline results across the case studies and emissions estimated with IPCC model are presented in Table 2. Sniffer data on individual dairy cow CH_4_ production differed by about 10% from those estimated by the IPCC Tier II model (IPCC, 2019). In contrast, sniffer data on CH_4_ for MB were almost identical to those estimated by the same IPCC model. Substantially, our results for dairy cows are in line with those obtained by other authors using different techniques. Ma et al. (2024) monitored CH_4_ emission in HF and BS cows using the respiration chamber, which is considered the most precise technique for benchmarking individual animal emissions, and showed an average production of 443 g CH_4_/d and an emission intensity of 16.2 g CH_4_/milk yield. In HF cows very similar to those in the present study for BW and milk production, Manzanilla-Pech et al. (2021) estimated the individual CH_4_ emissions using in vivo techniques and reported a production of 392 g CH_4_/d. Finally, Denninger et al. (2020) investigated the emissive levels of BS using laser methane detectors and, for animals with a BW between 570 and 740 kg, indicated a CH_4_ production ranging from 416 to 428 g/d. Knowledge about enteric CH_4_ emissions from buffaloes monitored through in vivo trials is limited, and mostly relies on indirect estimates or predictions derived from statistical models (Patra, 2014). Results of the present study were approximately 15% (Lanzoni et al., 2022) to 30% (Petrocchi Jasinski et al., 2025) lower than those reported in previous studies employing laser methane detectors. As reported in Table 2, the pipeline selected 60% and 68% of milkings for HF and BS cows, respectively, whereas only 35% of buffalo milkings met the pipeline criteria. The pattern and shape of detected peaks differed between cows and buffaloes (Figure 1), influencing both the number of identified peaks and the proportion of valid milkings. Throughout the pipeline, most milkings were filtered at the step requiring the presence of at least 3 peaks, a metric that has been shown to be consistent with respiration chamber records (Bell et al., 2020). Therefore, discarded milkings were considered less accurate and reliable. The CH_4_ peaks from buffalo eructation exhibited a sharp and narrow shape, characterized by only a few points defining the spike and poorly delineated sides. In contrast, peaks detected in cows showed a rapid increase in CH_4_ before the spike followed by a more gradual decline afterward. Despite similar acceptance of the AMS by both species, buffaloes tend to exhibit higher behavioral reactivity (Faugno et al., 2015), which may result in greater head movement and reduced proximity to the sniffer inlet. Our hypothesis is that behavioral aspects during AMS milking might affect the sniffer's efficiency in catching an animal's exhaled breath. However, head position was not directly monitored. Differences in CH_4_ peak patterns between species may reflect variations in ruminal anatomy, microbial populations, and feeding behaviors, leading to distinct gas production rates and total daily methane emissions (Bertoni et al., 2020). Additionally differences may not necessarily reflect species-specific traits, but could also be influenced by the age of monitored animals. In our dataset, the mean parity of buffaloes was 1.3, indicating that most animals were in their first lactation and may not have yet been fully accustomed to the milking system, unlike the HF and BS in this study. Our hypothesis requires confirmation through additional trials in other herds, especially for buffaloes because this represents the first evidence from enteric methane monitoring with a sniffer in buffalo. In recent years, several pipelines for processing sniffer data have been proposed, sharing the common objective of improving CH_4_ estimates on large-scale monitoring (L⊘vendahl et al., 2024; van Breukelen et al., 2025; Ryczek et al., 2026). Although these pipelines differ in implemented criteria, include procedures for background correction, peak detection, and filtering of unreliable visits. However, a systematic comparison of these pipelines is still needed. The proposed workflow enables the application to different farming systems and potential compatibility with sniffers of varying data resolution or with automatic feeder installations. However, some limitations inherent to the sniffer remain unresolved. Until now, the spatial distribution of CH_4_ and CO_2_ in air has not been considered; nevertheless, their different molar weights may have implications for sniffer recordings. Notably, CO_2_ has a larger molecular weight than air and may accumulate in the AMS feeder, creating localized concentrations that affect the background signal. Further research is needed to determine whether a feeder-specific background exists, composed by environmental background plus residual exhaled CO_2_, and whether this pattern may be affected by the sequential presence of animals in the AMS or by structural feed bin design. In this context, the use of the CH_4_:CO_2_ ratio presents several concerns for estimating breeding values due to the influence of multiple variables. The ratio may be affected by feed intake, energy efficiency, and energy balance, potentially favoring the selection of cows with less efficient metabolism, emitting more CO_2_ per unit of CH_4_, rather than with genuinely lower CH_4_ emissions (Schneider et al., 2025). In conclusion, the proposed pipeline provides a guided procedure that combines fragmented knowledge on sniffer data processing, demonstrating its application on 2 dairy cow breeds and buffaloes. This pipeline requires validation at a larger scale, involving other sniffer brands and monitored animals across different AMS systems, as well as cross-validation using other measurement technologies that provide CH_4_ emissions in grams per day. Broader application of this methodology, coupled with collaboration among sniffer users, is essential to building consistent, harmonized enteric emissions data and understanding CH_4_ and CO_2_ dynamics across breeds and species.

**Table 2:** Emission outcomes from the developed pipeline and from the Tier 2 IPCC model (IPCC, 2019) across the 3 case studies^1^

Item	Farm		
	HF	BS	MB
Valid milkings, %	60	68	35
CH_4_, g/d	416.7 ± 74.9	502.1 ± 158.4	279.0 ± 97.5
CO_2_, g/d	12,769 ± 624.3	10,735.8 ± 411.2	9,501.8 ± 957.5
CH_4_:CO_2_ ratio, vol/vol	0.09 ± 0.02	0.13 ± 0.04	0.08 ± 0.03
CH_4_ intensity, g CH_4_/kg ECM	13.3 ± 4.8	26.2 ± 10.6	28.7 ± 24.0
IPCC estimated CH_4_, g/d	457.6	467.6	276.5

1 IPCC values refer to herd-level estimates, whereas sniffer CH_4_, g/d represent averaged individual-level values. Reported variability (values after ±) represents SD.
